# 5-Hydroxymethyl-2-Furfural Oxidation Over Au/Ce_x_Zr_1-x_O_2_ Catalysts

**DOI:** 10.3389/fchem.2020.00461

**Published:** 2020-06-04

**Authors:** Cristina Megías-Sayago, Danilo Bonincontro, Alice Lolli, Svetlana Ivanova, Stefania Albonetti, Fabrizio Cavani, José A. Odriozola

**Affiliations:** ^1^Departamento de Química Inorgánica e Instituto de Ciencia de Materiales de Sevilla, Centro Mixto Universidad de Sevilla-CSIC, Seville, Spain; ^2^Dipartimento di Chimica Industriale “Toso Montanari, ” Università di Bologna, Bologna, Italy

**Keywords:** gold, 5-hydroxymethyl-2-furfural, oxidation, 2, 5-furandicarboxylic acid, support effect, Ce_x_Zr_1-x_O, CeO_2_, ZrO_2_

## Abstract

A series of gold catalysts supported on pure CeO_2_, ZrO_2_, and two different Ce-Zr mixed oxides have been prepared and tested in the 5-hydroxymethyl-2-furfural oxidation reaction. All catalysts show high catalytic activity (100% conversion) and important selectivity (27–41%) to the desired product i.e., 2,5-furandicarboxylic acid at low base concentration. Products selectivity changes with the support nature as expected, however, the observed trend cannot be related neither to gold particle size, nor to catalyst reducibility and oxygen mobility. An important relation between the FDCA selectivity and the support textural properties is observed, conducing to the general requirement for optimal pore size for this reaction.

## Introduction

The oxidation of 5-hydroxymethyl-2-furfural (HMF) to 2,5-furandicarboxylic acid (FDCA) is widely recognized as a potential process to replace petroleum-derived chemical, terephthalic acid, with a biorefinery-derived one (Zhang and Deng, 2015). Due to its chemical similarity, the polymerization of FDCA (concretely the dimethyl ester of FDCA) with ethylene glycol forms polyethylene furanoate (PEF), a plastic based polymer comparable to polyethylene terephthalate (PET) (Gandini et al., 2008). Certainly, recent studies have focused on the comparison of PEF and PET overall performances, revealing PEF's superiority in terms of physical, mechanical, and thermal properties (Burgess et al., 2014). The latter has motivated the scientific community to investigate FDCA production processes, mainly based on the efficient transformation of 5-hydroxymethyl-2-furfural (HMF). Most research focused on the catalytic screening of several systems, mainly based on noble metals, under different reaction conditions, being Ru (Nie et al., 2013; Yi et al., 2016), Pd (Siyo et al., 2014; Liu et al., 2015; Zhang et al., 2015), Pt (Siankevich et al., 2014; Ait Rass et al., 2015; Han et al., 2016), and Au (Lolli et al., 2015; Masoud et al., 2018; Megías-Sayago et al., 2018) the most reported. The HMF oxidation performances, logically, depend on the used metal, the support's chemical nature and the employed reaction conditions, with a special emphasis on the presence or absence of base (Zope et al., 2010; Pasini et al., 2011; Ardemani et al., 2015; Menegazzo et al., 2015; Du et al., 2018; Ferraz et al., 2018). Among the noble metals, gold has demonstrated to be alike to the others, more resistant to deactivation by overoxidation (Zhang and Deng, 2015) and sometimes even more active (Davis et al., 2011, 2012; Zhang and Deng, 2015).

**Scheme 1:**
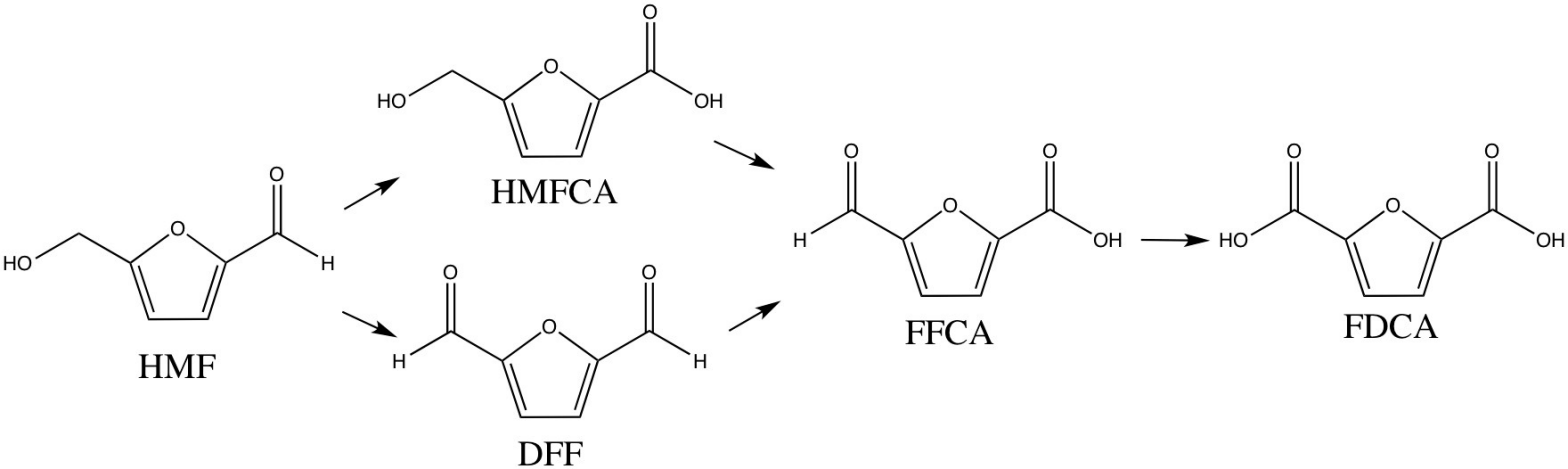
General reaction network for HMF oxidation.

The HMF molecule possesses two side chain functional groups, aldehyde and alcohol, and their cascade oxidation leads to the dicarboxylic acid, i.e., FDCA. The general reaction network (Scheme 1) includes 5-hydroxymethyl-2-furancarboxylic acid (HMFCA), 2,5-diformylfuran (DFF), 5-formyl-2-furancarboxylic acid (FFCA), and final 2,5-furandicarboxylic acid (FDCA). The use of gold orientate the HMF oxidation via HMFCA pathway, DFF is never detected, being the rate-limiting step the HMFCA to FFCA oxidation (Casanova et al., 2009; Albonetti et al., 2012; Lolli et al., 2015).

Interesting studies concerning mechanistic aspects have been carried out by Davis' group (Zope et al., 2010; Davis et al., 2012). They proved, by means of labeling experiments, that gold/water interfaces in presence of oxygen participate in the formation of hydroxyl ions from dioxygen and water, being the formed ions included in the reaction. Both hydroxyl species and HMF adsorb on gold surface without a direct support contribution in this mechanism. On the other hand, a recent study of our group (Megías-Sayago et al., 2018) reported a key role for the support surface in the rate-limiting step, particularly of support's Brönsted acidity and its participation in the reaction mechanism. Although reported as a key factor influencing the activity, the acidity is not the only one. The active sites access, reactive adsorption, product desorption, and selectivity are influenced by support's textural, redox, and electronic properties. There is actually a lack of studies relating all support properties to HMF conversion and FDCA yield over gold-based catalysts. That is why the present work aims to give more insights on the influence of the support's properties in the HMF oxidation reaction over a series of Au/Ce_x_Zr_1−x_O_2_ catalysts.

## Experimental

### Materials

HAuCl_4_ (Johnson Matthey), Ce(NO_3_)_3_·6H_2_O (Sigma Aldrich), and ZrN_2_O_7_·*x*H_2_O (Sigma Aldrich) were used as precursors for the preparation of catalyst series. The following products were used for the reaction: sodium hydroxide (Sigma Aldrich), 5-hydroxymethyl-2-furfural (AVA Biochem), 2,5-furandicarboxylic acid, 5-hydroxymethyl-2-furancarboxylic acid, 5-formyl-2-furancarboxylic acid, and 2,5-diformylfuran (the last three from Toronto Research Chemicals).

### Catalyst Preparation

Homemade supports were prepared according to coprecipitation method (Letichevsky et al., 2005), using the appropriate precursor quantities to obtain Ce-Zr mixed systems of 25 and 50 wt% in CeO_2_ (named as Ce25Zr and Ce50Zr, respectively). Zirconium precursor was dissolved in hot water and then mixed with cerium precursor under continuous stirring at pH 9, adjusted with NH_3_ (Panreac). Solids were dried at 110°C during 4 h and calcined in static air at 500°C for 1 h, with a heat ramp of 10°C/min. Analogous procedure was used for pure oxides, CeO_2_ and ZrO_2_ (named Ce and Zr, respectively). All the solids were powdered in an agate mortar with the fraction of 100–200 mm used for gold deposition. Gold (2 wt% nominal value) was deposited on the four different supports according to the Direct Anionic Exchange (DAE) method assisted by ammonia, proposed previously by Ivanova et al. (2004). In a typical procedure, a solution of the gold precursor (around 10^−4^ M) was heated to 70°C, and contacted with the support for 20 min. After that, a suitable amount of NH_3_ was added. The final solid was filtered, dried at 100°C overnight and calcined at 300°C for 4 h. This method was chosen for its relative reproducibility in loadings and particles size for supports with similar isoelectric point and chemical compositions.

### Characterization Techniques

XRD measurements were performed at room temperature on Panalitycal X'Pert Pro diffractometer, equipped with Cu anode. All diffractograms were recorded in 5–90°2θ range, with 0.05° step size and 300 s acquisition time.

BET specific surface areas and pore diameters, calculated by the BJH method, were obtained using a Micromeritics Tristar II equipment. Prior to the analysis, the samples were outgassed at 250°C in vacuum.

TEM micrographs were performed both with a HRTEM Jeol 2010F and a TEM/STEM FEI TECNAI F20 using a high-angle annular dark field (HAADF) imaging mode at 200 kV.

Gold contents were determined by X Ray Fluorescence (XRF) using Panalitycal AXIOS spectrometer with Rh tube of radiation.

Temperature programmed reduction experiments (TPR-H_2_) were carried out over ~50 mg of sample charged in a conventional U-shaped reactor under heating at constant rate of 10°C/min till 900°C under 50 mL/min certified 5% H_2_ in Ar gas mixture. H_2_ consumption was followed by TCD detector and quantified by using CuO (99.999%) standard.

For the Oxygen Storage Complete Capacity (OSCC) 100 mg of catalyst were loaded into the same U-shaped quartz reactor. Every sample was pretreated in 50 mL/min He flow at 300°C for 2 h and after cooled down to the desired temperature. For each temperature, 10 O_2_ pulses of 1 mL were injected every 2 min. After that, the sample was submitted to 10 equivalent CO pulses. The OSCC is obtained from the total CO_2_ produced in all CO pulses. The sample was then degassed during 10 min in a He flow and subjected to a new series of oxidizing pulses (ten O_2_ pulses) and subsequently to six alternating pulses (CO–O_2_-CO–O_2_-CO–O_2_). The OSC is determined by the average amount of CO_2_ per pulse formed after the first CO pulse of the alternated ones. This method is based on that proposed by Kacimi et al. (1993) and Royer and Duprez (2011). The gas composition at the exit of the reactor was analyzed by a mass spectrometer PFEIFFER Vacuum PrismaPlus controlled by Quadera® software.

### Catalytic Tests

The oxidation of HMF was performed in an autoclave reactor of 100 mL of capacity, provided with a mechanical stirrer (0–600 rpm) and measurement tools for temperature and pressure. The reactor was charged with an aqueous solution of HMF (~25 mL), the necessary amount of NaOH and the catalyst in a HMF:Au:NaOH of 1:0.01:2. Before the test, the reactor was purged two times with pure O_2_ (10 bar) and finally pressurized to 10 bar. Temperature was increased to 70°C and the reaction mixture was stirred at ~400 rpm for 4 h. The reaction starts (*t* = 0) when the temperature reached 70°C (about 10 min). After 4 h, the reactor was cooled down to room temperature in an ice bath and the reaction mixture centrifuged and filtered. A sample was taken and diluted before analysis with an Agilent Infinity 1260 liquid chromatograph equipped with an Aminex HPX-87H 300 mm × 7.8 mm column using 0.005 M H_2_SO_4_ as eluent. Conversion, selectivity and yield were calculated from peak areas, after calibration using reference commercial samples, according to the following equations:

(1) $$\begin{array}{r} Conversion\;\left(\%\right)=\;\frac{{\left[HMF\right]}_{I}-{\left[HMF\right]}_{F}}{{\left[HMF\right]}_{I}}\;x\;100 \end{array}$$

(2) $$\begin{array}{r} FDCA\;Selectivity\;\left(\%\right)=\;\frac{FDCA\;moles}{{HMF\;moles}_{I}-{HMF\;moles}_{F}}x\;100 \end{array}$$

(3) $$\begin{array}{r} FDCA\;Yield\;\left(\%\right)=\;\frac{Conversion}{100}x\;Selectivity \end{array}$$

## Results and Discussion

### Catalysts Characterization

Table 1 summarizes the chemical composition of the samples estimated by X-Ray Fluorescence (XRF), the gold particles sizes estimated by TEM and the calculated gold dispersions. The composition of the mixed oxides results to be very close to all targeted values, being 44.7 and 24.2 wt% CeO_2_ for Ce50Zr and Ce25Zr samples, respectively. Regarding the gold loadings, out of AuCe sample that shows unexpectedly high amount, all other samples demonstrate good approximation to the targeted values. Considering that the gold deposition was carried out with the same procedure for all the samples (same precursor to support ratio), the doubled Au loading for the AuCe sample indicates analysis problem or a probable matrix effect. The same was observed for a series of Au/CeZr catalysts prepared over commercial supports with similar composition, reported elsewhere (Megías-Sayago et al., 2018).

**Table 1:** Chemical compositions and dispersion of the selected solids.

**Sample**	**wt% Au**	**wt% CeO_**2**_**	**wt% ZrO_**2**_**	**Gold particles size, nm**	**Dispersion, %**
Ce		100			
AuCe	4	96		3.7 ± 1.3	35
Ce50Zr		44.7	55.2		
AuCe50Zr	2.5	45.1	52.4	2.9 ± 0.8	44
Ce25Zr		24.2	75.8		
AuCe25Zr	2.5	19.4	78.1	2.1 ± 0.4	57
Zr			100		
AuZr	2.3		97.7	3.1 ± 1.5	41

**Figure 1:**
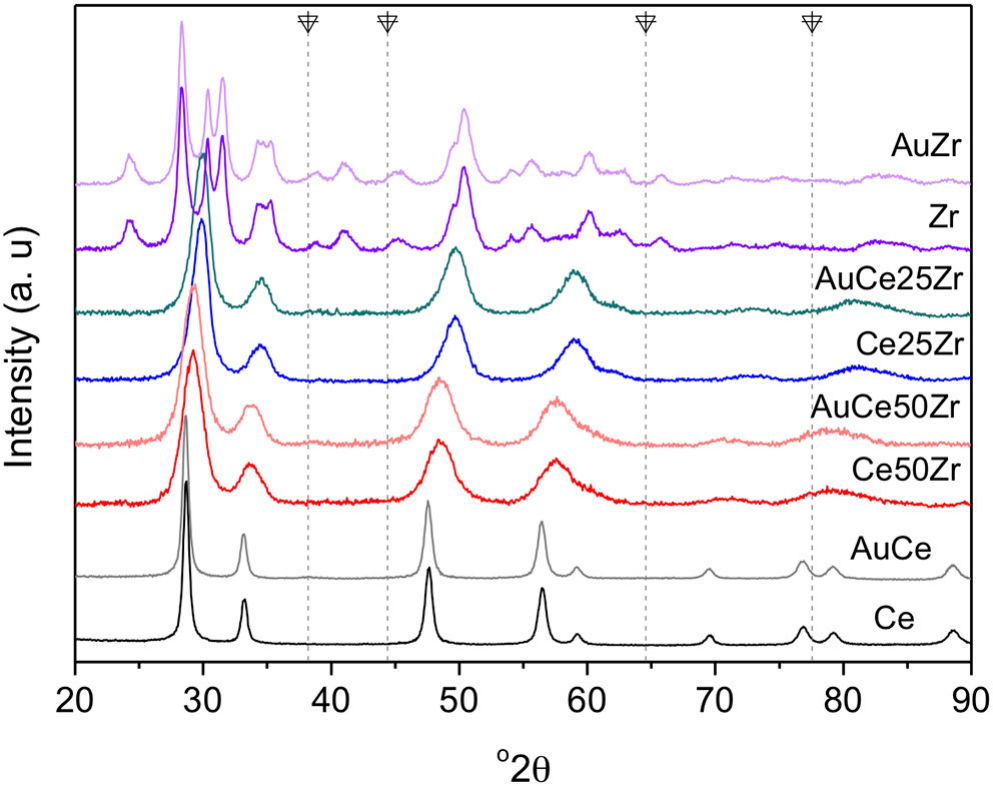
X-ray diffraction patterns of Ce_x_Zr_1−x_ O_2_ supports and its corresponding gold catalysts. Main gold diffractions are marked as 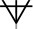.

CeO_2_ sample presents a set of intense diffractions (Figure 1) corresponding to the cubic fluorite structure (ICSD #00-034-0394). For the mixed oxides, the zirconia introduction induces two evident effects. Firstly, the formation of homogeneous solid solution, predicted based on the diffraction pattern shift: Ce (111) shifts from 28.7° 2Θ to 29.3 and 29.8 for Ce50Zr and Ce25Zr samples, respectively. All binary systems present typical diffraction patterns of Ce_x_Zr_1−x_O_2_ solid solutions (Thammachart et al., 2001; Gutiérrez-ortiz et al., 2004) with absence of pure CeO_2_ and ZrO_2_ segregated phases. After 50% zirconia addition, the diffractions of CeO_2_ fluorite structure move to higher angles, because of ceria lattice shrinkage, caused by the substitution of Ce^4+^ (0.098 nm ionic radii) with smaller Zr^4+^ cations (0.084 nm) (Gutiérrez-ortiz et al., 2004). This effect confirms the formation of a solid solution. The introduction of 75% zirconia, however, induces even higher diffraction shifts attributed to the zirconia lattice expansion due, in this case, to the replacement of Zr^4+^ cations with bigger Ce^4+^ cations. Actually and according to the literature (Gutiérrez-ortiz et al., 2004) Ce50Zr sample has a cubic structure (ICSD #00-028-0271) whereas Ce25Zr sample presents tetragonal one (ICSD #01-080-0785). Pure ZrO_2_ solid shows the presence of both, cubic (ICSD #00-003-0640) and monoclinic (predominant, ICSD #00-007-0343) structures. Furthermore, the second effect induced by Zr introduction concerns particles size diminution, evidenced by the diffractions broadening in zirconia containing samples. Reina et al. (2013) observed similar effect with the introduction of defects for the cerium iron mixed oxides. This effect could be ascribed to the different nucleation rate between ZrO_2_ and CeO_2_-rich phases. Regarding gold catalysts, no diffraction attributed to gold (vertical dashed lines) appears suggesting small gold particles size.

**Figure 2:**
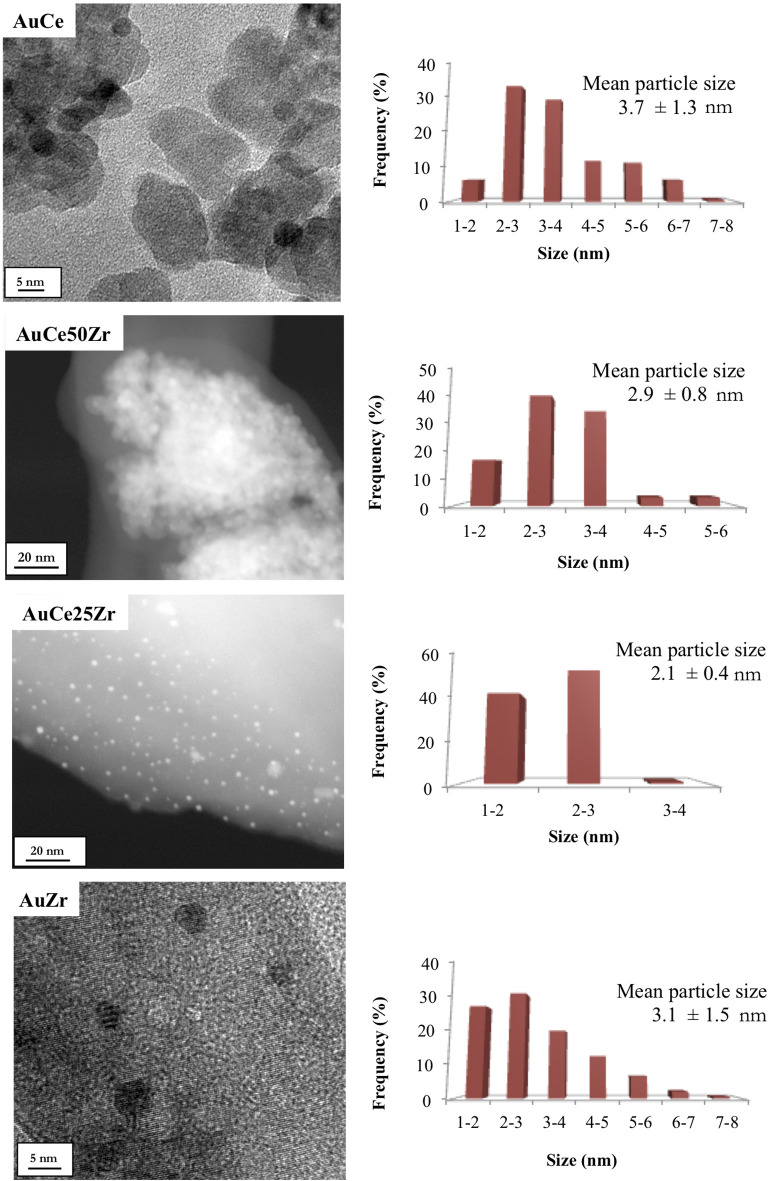
TEM/STEM images and particle size distributions of prepared samples.

Indeed, a small average gold particle size is estimated using High-resolution transmission electron microscopy (HR-TEM) combined with high-angle annular dark-field scanning transmission electron microscopy (HAADF-STEM). Due to the similar atomic weight of gold and cerium, their individual identification often becomes a difficult task. For this reason, both techniques were combined to get the best possible contrast. Representative micrographs and particle size distributions are presented in Figure 2.

The mean gold particle size ranges 2.1–3.7 nm (Table 1) with a typical Gaussian like distribution. On pure CeO_2_ and ZrO_2_ the supported gold particles present higher average size and larger distribution, while for the mixed oxides the opposite is observed. The presence of higher Zr content seems to promote gold average particle size decrease. A plausible explication of this fact is the increase of the defect population in the mixed samples and facilitated gold nucleation over the defects sites (Hernández et al., 2010; Menegazzo et al., 2015).

Table 2 compares the specific surface area, average pore size and pore volume of Au-supported samples.

**Table 2:** Textural properties of the studied Au catalysts.

**Sample**	**S_**BET**_ (m^**2**^/g)**	**Average pore size (nm)**	**Pore volume (cm^**3**^/g)**
AuCe	49	9.9	0.143
AuCe50Zr	82	3.1	0.072
AuCe25Zr	81	3.6	0.095
AuZr	56	3.7	0.076

All prepared catalysts are mesoporous solids with areas ranging from 49 to 82 m^2^/g, being that of pure oxides a half of the area of the mixed oxides. The solids present pore sizes around 3 nm exception made by the AuCe sample with considerately superior size (9.9 nm). The differences are also noticeable regarding the pore volume distributions, highest the average pore size highest the corresponding pore volume. We can suppose that the higher surface area for low pore size and volume for the mixed oxides corresponds to the interparticular surface, and lower the particle size (evidenced by XRD) higher the area and lower the pore size.

**Figure 3:**
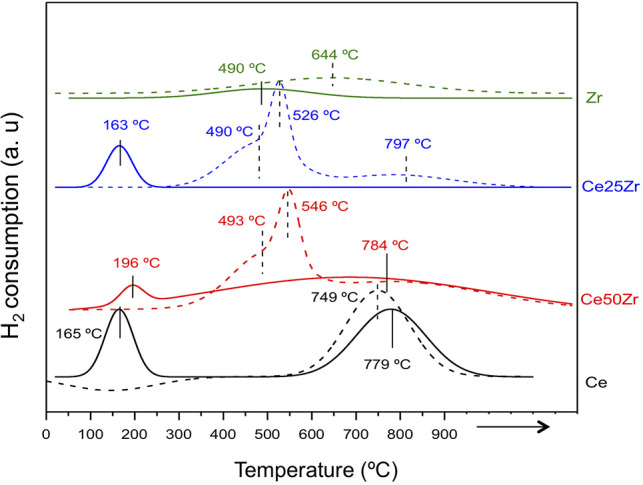
TPR-H_2_ profiles of the supports (dashed lines) and their corresponding gold catalysts (full lines).

The catalyst's redox properties were investigated by means of temperature-programmed reduction (TPR). H_2_ consumption profiles as a function of the temperature for the prepared series are presented in Figure 3. Bare ceria sample shows only one zone of reduction centered at 749°C, assigned to the reduction of surface ceria. A typical ceria samples reduction profile is characterized by two zones, surface ceria at lower temperatures and bulk ceria at higher temperatures (Gutiérrez-ortiz et al., 2004). The appearance of only one reduction zone suggests a probable bulk ceria reduction at higher temperatures (>900°C) that cannot be registered by our equipment which is consistent with some literature reports (Kaspar et al., 1999).

In contrast, the TPR profiles of the mixed oxides are much complex. The introduction of ZrO_2_ into ceria and vice versa leads to lattice deformation, as revealed by XRD, and as a consequence, the reduction process is not limited to the surface but also involves more easily the bulk (Gutiérrez-ortiz et al., 2004). The oxide reduction (understood as loss of oxygen atoms) is strongly influenced by the oxygen mobility, utterly boosted by the presence of zirconia. Therefore, the mixed oxides reduction zones shift to lower temperatures and the reduction percentage increases. Now, it is possible to distinguish three reduction zones, attributed to oxygen atoms from different layers stuck between surface and bulk. As for ZrO_2_ profile no clear reduction process is detected in agreement with the literature (Kaspar et al., 1999), although some hydrogen consumption is registered, most probably caused by hydrogen adsorption/desorption processes (Kondo et al., 1990).

When gold is introduced to the systems the reduction process is apparently more complete (higher area of the zones) and shifts to lower temperature. Presumably the hydrogen mobility is facilitated by the metal and the reduction rate rises significantly in the presence of gold (Jacobs et al., 2004). In a similar manner, the low temperature reduction zone is assigned to the noble metal promoted ceria surface reduction and the high temperature reduction process to the ceria bulk reduction. The AuCe25Zr sample shows only one reduction event centered at 163°C. It appears that the redox behavior of the mixed oxides depends on zirconia content, higher the content narrower the reduction zone at lower temperatures.

The reducibility percentages (RP) are calculated according to the following equation:

(4) $$\begin{array}{r} RP=\;\frac{E_{HC}}{T_{HC}}\;x\;100 \end{array}$$

**Figure 4:**
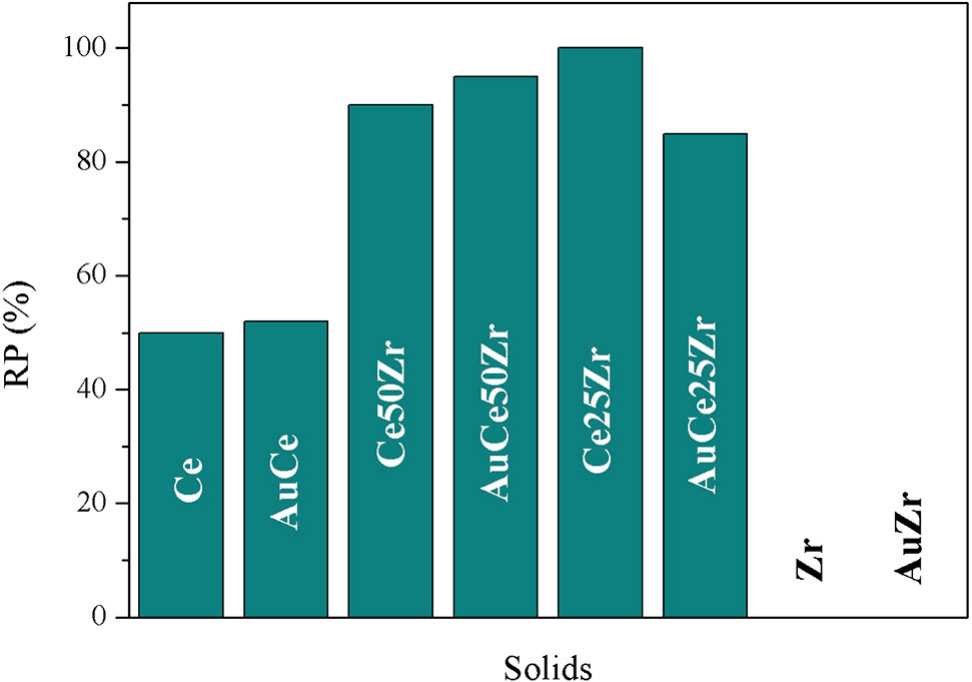
Reducibility percentage (RP) of the prepared solids.

The RP relates the experimentally measured H_2_ consumption (E_HC_) to the total theoretical H_2_ consumption (T_HC_). The T_HC_ depends on the oxidation state and the number of considered reducible species. For all samples, Ce^4+^ species are considered reducible to Ce^3+^ species (Equation 5), whereas the Zr^4+^ remain irreducible under H_2_ flow. As for gold, due to the auto-reduction capability of gold species during the calcination process, gold is presumably metallic, and therefore non-reducible species. The results are presented in Figure 4.

(5) $$\begin{array}{r} 2\;CeO_{2}+\;H_{2}\to Ce_{2}O_{3}+\;H_{2}O. \end{array}$$

The samples of pure ceria show 50% of total reduction degree in the temperature range considered in this study. However, the mixed oxide samples present higher RP due most probably to the higher oxygen mobility. The RP decreases as following:

$$\begin{array}{l} \text{AuCe}50\text{Zr}>\text{AuCe}25\text{Zr}>\text{AuCe}>\text{AuZr} \end{array}$$

### Catalytic Tests

**Figure 5:**
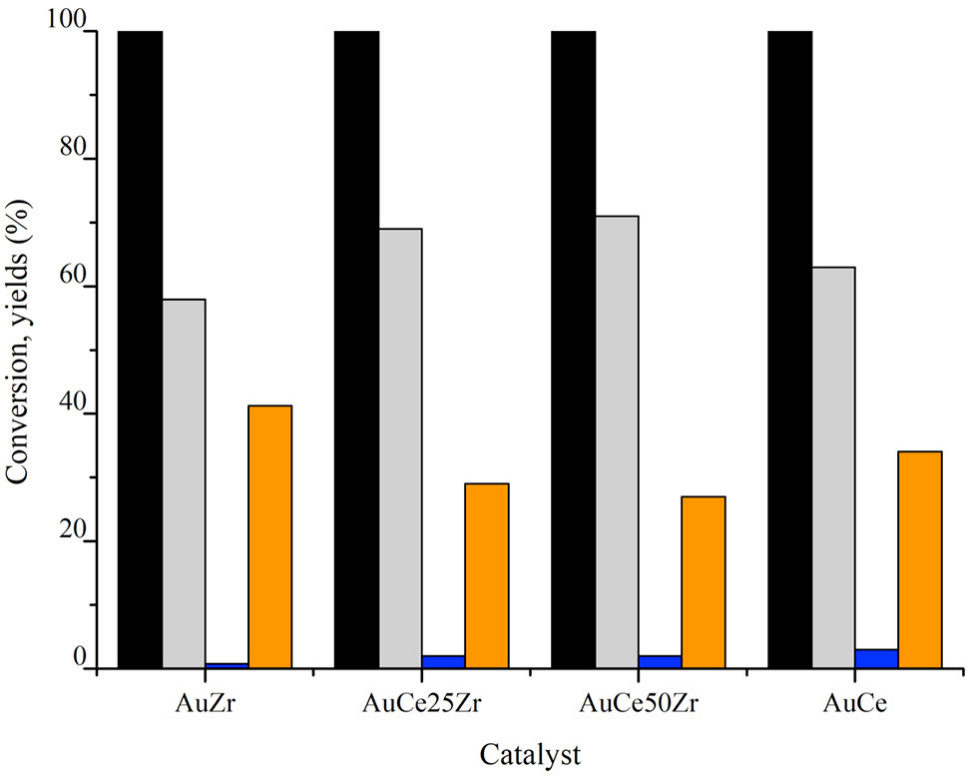
Catalytic activity of the prepared samples. Reaction conditions: 4h, 70°C, 10 bar O_2_, HMF:Au:NaOH 1:0.01:2, Legend: HMF conversion (■), HMFCA yield (■),FFCA yield (■), FDCA yield (■).

Before testing the catalysts, the blank tests of the supports resulted in the formation only of by-products, due to HMF degradation in the presence of NaOH. The supports were not able to convert catalytically HMF to the desired product. On the contrary, the presence of gold promoted the HMF conversion and its selective oxidation to 2,5-furandicarboxylic acid (FDCA) and 5-hydroxymethyl-2-furancaboxylic acid (HMFCA). The latter is the first reaction intermediate as shown in Scheme 1, which converts lately to 5-formylfuran-2-carboxylic acid (FFCA) and finally to FDCA. FFCA is detected only in negligible amounts due to its rapid conversion to the final product. In fact, the rate-limiting step of the reaction is the alcohol group oxidation (Siyo et al., 2014; Albonetti et al., 2015). This is the preferred pathway for HMF oxidation in presence of NaOH, diformylfuran (DFF) is never formed (Lolli et al., 2015). All catalysts showed nearly 100% of HMF conversion (Figure 5), AuZr and AuCe exhibiting higher FDCA yield than that of gold supported over mixed oxides.

The catalytic behavior could be influenced by the presence of CeO_2_ and its redox nature, acidity, and textural properties, or by gold dispersion. Considering ZrO_2_ irreducible, the catalysts' oxygen mobility should arise from CeO_2_ and collaborative CeO_2_-Au or Ce-Zr-O interactions.

The oxygen storage ability can be quantified by means of oxygen storage capacity (OSC) and oxygen storage complete capacity (OSCC) measurements. The OSCC offers information about the total oxygen species available within the sample and is estimated from the formed CO_2_ during 10 consecutive CO pulses. On the other hand, OSC corresponds to the most accessible species and is calculated from the average of CO_2_ species formed under sample's exposure to consecutive CO – O_2_ pulse sequences. The redox processes involved in the oxygen storage are:

$$\begin{array}{l} \text{Ce}{\text{O}}_{2}+xCO\to CeO_{2-\text{x}}+xCO_{2}\;\left(reduction\right)\\ 2\text{Ce}{\text{O}}_{2-\text{x}}+O_{2}\to 2CeO_{2}\;\left(oxidation\right)\\ \text{C}{\text{e}}_{\text{x}}Zr_{1-\text{x}}O_{2}+\delta CO\to Ce_{\text{x}}Zr_{1-\text{x}}O_{2-\text{δ}}+\delta CO_{2}\;\left(reduction\right)\\ \text{C}{\text{e}}_{\text{x}}Zr_{1-\text{x}}O_{2-\text{δ}}+O_{2}\to Ce_{\text{x}}Zr_{1-\text{x}}O_{2}\;\left(oxidation\right) \end{array}$$

**Figure 6:**
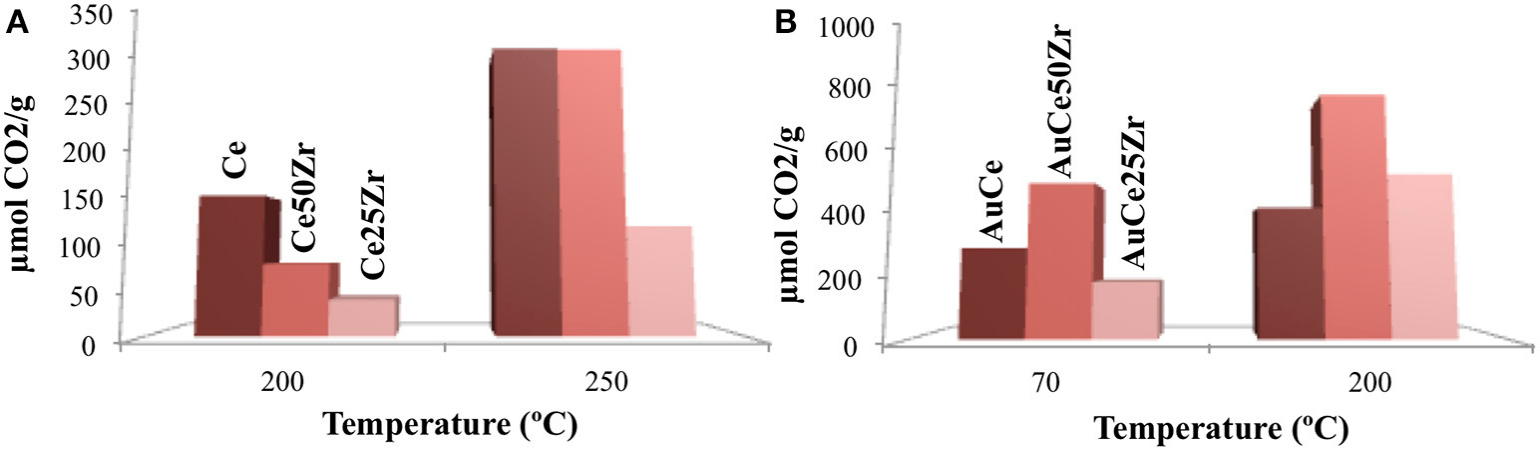
OSCC for the supports (**A**, 200 and 250°C) and for the catalysts (**B**, 70 °C and 200°C).

The OSCC measurements expressed in μmol formed CO_2_ are presented in Figure 6. For the bare supports, the measured temperatures are 200 and 250°C, whereas for the catalysts are 70°C (the reaction temperature) and 200°C (a temperature that allows support/catalyst comparison). As a general trend, the increase of the temperature increases the OSCC value. The bare support follows the O_2_ mobility trend Ce > Ce50Zr > Ce25Zr at 200°C and Ce50Zr ~ Ce>> Ce25Zr at 250°C. Zirconia presence improves ceria's oxygen mobility, being the Ce50Zr the optimal composition, in agreement with TPR analysis, where zirconium introduction caused an increase of the reducibility of the system.

The oxygen mobility is promoted even more in the presence of gold, being noticeable even at 70°C (reaction temperature). Here the tendency differs from that of supports, being AuCe50Zr > AuCe > AuCe25Zr the one at low temperatures and AuCe50Zr > AuCe25Zr > AuCe at high temperatures. The latter is consistent with previous studies, in which it was demonstrated that the oxygen storage capacity depends on zirconia content and a maximal value is obtained for 25–50 mol% of Zr (Kaspar et al., 1999). Considering only the OSCC at the reaction temperature (70°C), the oxygen mobility does not correspond to the observed activity. If we consider the information received by the OSC measurements, the number of the atomic oxygen layers (NL) directly involved in the process can be calculated, according to Equation 6.

(6) $$\begin{array}{r} NL=\;\frac{OSC_{experimental}}{OSC_{surface}}. \end{array}$$

where OSC_experimental_ is the that obtained experimentally and OSC_surface_ accounts for the theoretically reducible oxygen on the surface. The OSC_surface_ is calculated by equation 7.

(7) $$\begin{array}{r} OSC_{surface}\;\mu molCO_{2}g^{-1}=\;N_{0}\;x\;S_{BET}\;x\;\frac{1}{N_{A}}\;x\frac{1}{a^{2}}\;x\;10^{6}. \end{array}$$

where *S*_*BET*_ is the specific surface area of the sample, *N*_*A*_ is Avogadro's number, *a* is the ceria lattice parameter (5.413 Å) and *N*_0_ is the number of oxygen atoms of the ceria lattice that participate in the process. This number depends on the exposed CeO_2_ lattice planes. In this study an average of the exposed oxygen from the (001), (110), and (111) faces has been assumed for the calculations resulting in a *N*_0_ value of 1. Indeed, only one oxygen is participating in both redox processes expressed above. It is worth to clarify that NL <1 means that only oxygen from the surface is playing a role in the reduction process while NL >1 indicates the participation of bulk oxygen.

The calculations of OSC are based on the methodology proposed by Madier et al. (1999). More precisely, it is considered that (i) only oxygen atoms bonded to cerium participate in the storage process; (ii) the surface is homogeneous (iii) only one of four oxygen atoms is involved in the storage (CeO_2_ Ce_2_O_3_ + “O”); and (iv) null gold metal contribution to the reduction, e.g., the gold metal cannot re-oxidize. For the OSC theoretical calculations, the number of surface oxygen atoms and BET area of each sample are considered.

For the supports (Table 3), similar to OSCC higher the temperature higher the fraction of layer (and therefore the OSC) involved in the reduction. Similarly to OSCC, the OSC decreases in order Ce > Ce50Zr > Ce25Zr at 200 and 250°C. The addition of gold changes dramatically the oxygen mobility at 200°C (Table 4) as well as the OSC tendency. At the reaction temperature, the oxygen dynamics is 16 times higher for AuCe50Zr that for the support at 200°C. The oxygen involved in the OSC is the surface oxygen (NL < 1) for the majority of samples no matter the temperature, exception made by AuCe50Zr at 200°C where the bulk oxygen also participates.

**Table 3:** Oxygen storage capacity (OSC), expressed as (μmol CO_2_/g), and number of oxygen layers (NL) for the supports as a function of the temperature.

**Sample**	**OSC 200^**°**^C**	**OSC 250^**°**^C**	**NL 200^**°**^C**	**NL 250^**°**^C**
Ce	16.40	67.97	0.05	0.21
Ce50Zr	6.39	28.96	0.02	0.09
Ce25Zr	3.57	14.89	0.01	0.01

**Table 4:** Oxygen storage capacity (OSC), expressed as (μmol CO_2_/g), and number of oxygen layers (NL) for the catalysts as a function of the temperature.

**Sample**	**OSC 70^**°**^C**	**OSC 200^**°**^C**	**NL 70^**°**^C**	**NL 200^**°**^C**
AuCe	68.57	103.03	0.21	0.32
AuCe50Zr	99.23	332.68	0.32	1.07
AuCe25Zr	58.20	124.88	0.19	0.40

The trend found for oxygen mobility AuCe50Zr > AuCe > AuCe25Zr, differs from the activity relation. Therefore, although the ability of the support to transfer oxygen could influence the activity, is neither the only factor involved nor the most important one. It is logical to consider that the catalytic activity is affected by a combination of other factors.

The chemical composition of the samples is very similar to a series of catalysts prepared over commercial supports in our previous study (Megías-Sayago et al., 2018). But the activity order in this study do not repeat the order in the previous study, and lower yields of FDCA are produced. If we suppose that, their acid properties are comparable (same Ce/Zr ratio) the difference in activity could be due either to a difference in gold particle size or to catalysts textural properties.

**Figure 7:**
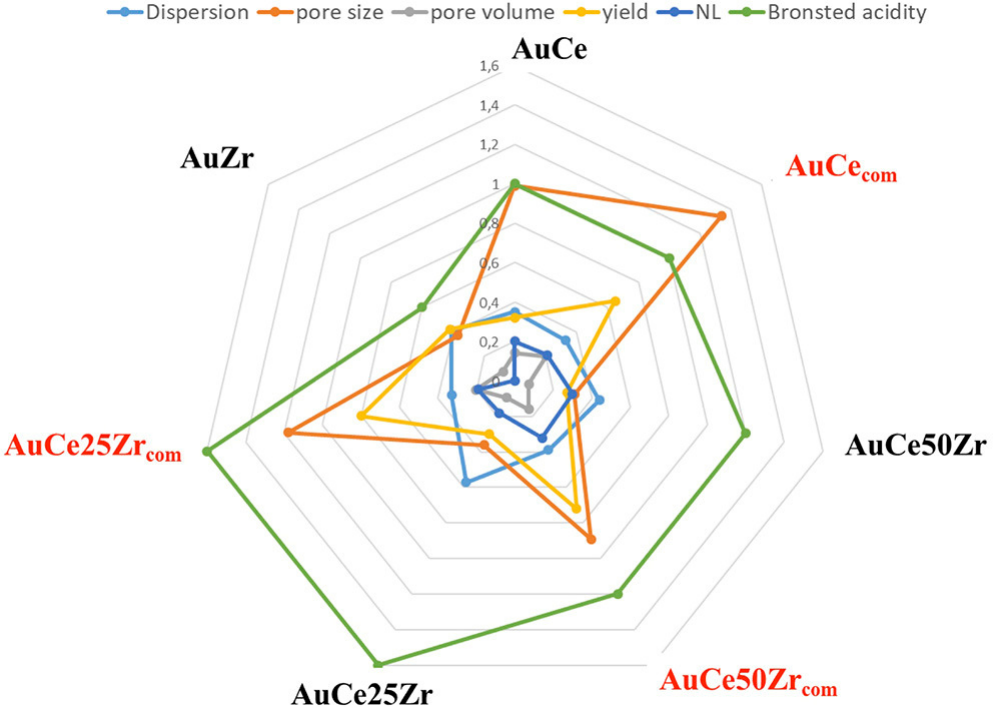
Multiple parameters plot over the as prepared series and a series over commercial supports (Megías-Sayago et al., 2018).

If we compare both series (over commercial Megías-Sayago et al., 2018 and homemade supports) considering dispersion, pore size and volume, acidity, redox properties, and FDCA yield we should be able to conclude on the parameter that influences the activity in greater extent (Figure 7). The data used for this figure are listed in supporting information (Table S1).

The dispersion of gold and the redox properties of the supports (expressed as NL number) do to not fit very well the observed yields, however, the acidity and especially the textural properties, pore size, and volume suits the observed trend. The samples separate in two groups, the commercial with higher pore diameter producing higher FDCA yields, and the samples from this study, with half of pores diameter and lower FDCA yield. It is very interesting to underline that the AuCe sample from the actual series presents higher pore size and as a consequence present activity closer to the group of the commercial samples.

The obtained relation suggests that (i) gold nanoparticles may be located in the pores (ii) the FDCA production occurs in the pores. Both suggestions imply that in small pores solids diffusional limitations could appear.

Deducing which diffusion step is the limiting one is very difficult, due to the multiple mass-transfer processes involved in the reaction, such as (i) the oxygen dissolution in the HMF solution (gas-liquid transfer); (ii) the oxygen and reactants diffusion from the liquid bulk toward the outer surface of the catalyst grain (external diffusion); (iii) the transfer of the substrates from the outer surface inside the catalyst pores toward active sites of the reaction (internal reactant diffusion) and (iv) the transport of the reaction product from the pores to the outer surface of the catalyst grain (internal product diffusion).

**Figure 8:**
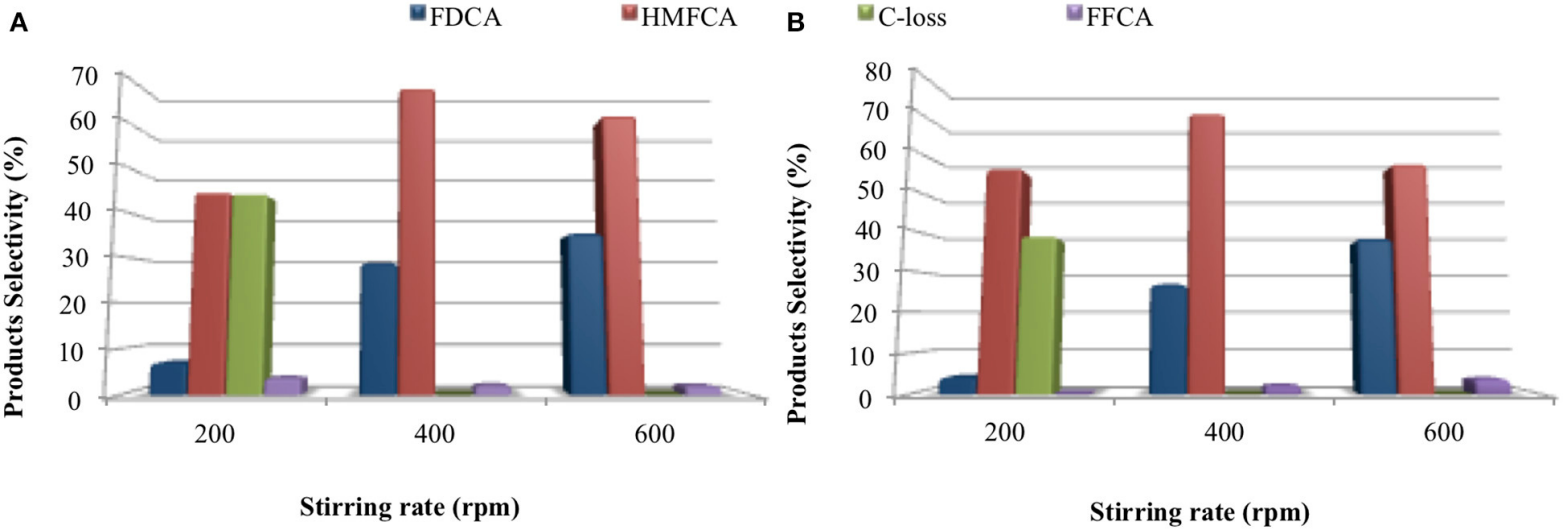
Stirring rate dependence of FDCA yield. **(A)** AuCe50Zr and **(B)** AuCe25Zr. Reaction conditions: HMF:Au:NaOH molar ratio 1:0.01:2, 10 bar O_2_, 70°C.

In order to verify the presence of external diffusional limitations, different experiments were carried out by changing the stirring rates, at 200 and 600 rpm over both AuCe50Zr and AuCe25Zr samples (Figure 8).

In both cases the FDCA yield decreases at 200 rpm (5%), resulting also in an important carbon loss of 40–45%. It seems that the low stirring rate prevents the proper diffusion of the HMF molecule to the active sites, being promoted the HMF degradation (via Cannizzaro reaction), resulting in byproducts formation and low carbon balance. Increasing the stirring rate to 600 rpm causes a slight increase of FDCA yield. However, the yields of the FDCA are still lower than those over commercial supports suggesting internal diffusion being present and indicating that an optimal pore size is necessary for the reaction (being the minimum size situated around 12 nm).

It has been demonstrated previously, that the surface Brönsted acidity of gold supported on Ce_x_Zr_1−x_O_2_ oxides plays a pivotal role in the reaction mechanism, being extremely beneficial for the FDCA production. We can assume that increasing the Zr/Ce ratio increases the number of surface hydroxyl groups, deprotonated in basic medium, and also promotes the formation of the alkoxy intermediate of HMFCA, favoring FFCA formation and, therefore, the total oxidation toward FDCA (Megías-Sayago et al., 2018). However, for this mechanism to occur the distribution of the support's non-isolated Brönsted sites situated in the vicinity of gold metal sites have to be fully accessible for adsorption/desorption which is only possible for specific pore size and volume. It seems that 12 nm pore size is the minimum size needed to avoid any of the mass-transfer processes involved, being possible to establish it as a general requirement for the liquid phase HMF oxidation.

## Conclusions

A series of gold catalysts supported on metal oxides have been prepared and tested in HMF oxidation reaction. All catalysts exhibit high catalytic activity (100% HMF conversion) and important selectivity to FDCA. The bare supports do not participate in the reaction; only byproducts via Cannizzaro reaction are formed. However, when gold is present the HMF conversion reaches 100% without byproducts formation. Products distribution changes with the support change as expected, however, the observed trend cannot be related to gold particle size or support redox properties. The Brönsted acidity of the samples is very important for the mechanism of reaction, however the reaction is limited by the catalysts pore size and a minimum pore size of 12 nm is established to avoid internal diffusional limitations during the oxidation process.

## Data Availability Statement

The raw data supporting the conclusions of this article will be made available by the authors, without undue reservation, to any qualified researcher.

## Author Contributions

CM-S: Conceptualization, methodology, data curation, formal analysis, visualization, investigation, writing-original draft, writing-review, and editing. DB: Methodology, data curation, and formal analysis. AL: Supervision, validation, and investigation. SI: Supervision, conceptualization, methodology, data curation, formal analysis, visualization, investigation, writing-review, and editing. SA and FC: Supervision and validation. JO: Supervision, validation, and funding acquisition.

## Conflict of Interest

The authors declare that the research was conducted in the absence of any commercial or financial relationships that could be construed as a potential conflict of interest.
