# Supplementary material for: 5-Hydroxymethyl-2-Furfural Oxidation Over Au/CexZr1-xO2 Catalysts
Source: Front Chem. 2020 Jun 4;8:461. doi: 10.3389/fchem.2020.00461 (PMC7287476; doi:10.3389/fchem.2020.00461)
Supplement: Supplementary file 1 [file Table_1.docx]

|  | **Dispersion % *10^-2^** | **Pore size, nm*10^-1^** | **Pore volume, cm^3^/g** | **FDCA yield, % *10^-2^** | **NL** | **Brönsted acidity, IR shift *10^-1^** |
| --- | --- | --- | --- | --- | --- | --- |
| AuCe | 0.35 | 0.99 | 0.143 | 0.32 | 0.2 |  |
| AuCe_com_ | 0.33 | 1.34 | 0.2 | 0.65 | 0.21 | 1 |
| AuCe50Zr | 0.44 | 0.31 | 0.072 | 0.27 | 0.3 |  |
| AuCe50Zr_com_ | 0.39 | 0.89 | 0.161 | 0.72 | 0.32 | 1.2 |
| AuCe25Zr | 0.57 | 0.36 | 0.095 | 0.3 | 0.18 |  |
| AuCe25Zr_com_ | 0.33 | 1.18 | 0.201 | 0.8 | 0.19 | 1.6 |
| AuZr | 0.41 | 0.37 | 0.0759 | 0.42 | 0 | 0.6 |

**Supporting Information**

**5-hydroxymethyl-2-furfural oxidation over Au/Ce_x_Zr_1-x_O_2_ catalysts**

C. Megías-Sayago^1^, D. Bonincontro^2^, A. Lolli^2^, S. Ivanova^1^, S. Albonetti^2^, F. Cavani^2^, J. A. Odriozola^1^

^1^Departamento de Química Inorgánica e Instituto de Ciencia de Materiales de Sevilla, Centro mixto Universidad de Sevilla-CSIC, Américo Vespucio 49, 41092, Seville, Spain

^2^Dip. di Chimica Industriale “Toso Montanari”, Università di Bologna, Viale Risorgimento 4, 40136 Bologna (BO), Italy

Table S1. Summary of available data from [1] and present work.

[1] C. Megías-Sayago, K. Chakarova, A. Penkova, A. Lolli, S. Ivanova, S. Albonetti, F. Cavani, J.A. Odriozola, ACS Catal. 8 (2018) 11154−11164.
